# Potential of human umbilical cord blood mesenchymal stem cells to heal damaged corneal endothelium

**Published:** 2012-03-02

**Authors:** Nancy C. Joyce, Deshea L. Harris, Vladimir Markov, Zhe Zhang, Biagio Saitta

**Affiliations:** 1 Schepens Eye Research Institute, Boston, MA; 2 Department of Ophthalmology, Harvard Medical School, Boston, MA; 3 Department of Cell Biology, University of Medicine and Dentistry of New Jersey-School of Osteopathic Medicine, Stratford, NJ; 4 Center for Biomedical Informatics, The Children's Hospital of Philadelphia, Philadelphia, PA

## Abstract

**Purpose:**

To test the feasibility of altering the phenotype of umbilical cord blood mesenchymal stem cells (UCB MSCs) toward that of human corneal endothelial cells (HCEC) and to determine whether UCB MSCs can “home” to sites of corneal endothelial cell injury using an ex vivo corneal wound model.

**Methods:**

RNA was isolated and purified from UCB MSCs and HCECs. Baseline information regarding the relative gene expression of UCB MSCs and HCEC was obtained by microarray analysis. Quantitative real-time PCR (q-PCR) verified the microarray findings for a subset of genes. The ability of different culture media to direct UCB MSCs toward a more HCEC-like phenotype was tested in both tissue culture and ex vivo corneal endothelial wound models using three different media: MSC basal medium (MSCBM), a basal medium used to culture lens epithelial cells (LECBM), or lens epithelial cell-conditioned medium (LECCM). Morphology of the MSCs was observed by phase-contrast microscopy or by light microscopic observation of crystal violet-stained cells. Immunolocalization of the junction-associated proteins, zonula occludins-1 (ZO1) and N-cadherin, was visualized by fluorescence confocal microscopy. Formation of cell-cell junctions was tested by treatment with the calcium chelator, EGTA. A second microarray analysis compared gene expression between UCB MSCs grown in LECBM and LECCM to identify changes induced by the lens epithelial cell-conditioned culture medium. The ability of UCB MSCs to “home” to areas of endothelial injury was determined using ZO1 immunolocalization patterns in ex vivo corneal endothelial wounds.

**Results:**

Baseline microarray analysis provided information regarding relative gene expression in UCB MSCs and HCECs. MSCs attached to damaged, but not intact, corneal endothelium in ex vivo corneal wounds. The morphology of MSCs was consistently altered when cells were grown in the presence of LECCM. In tissue culture and in ex vivo corneal wounds, UCB MSC treated with LECCM were elongated and formed parallel sheets of closely apposed cells. In both tissue culture and ex vivo corneal endothelial wounds, ZO1 and N-cadherin localized mainly to the cytoplasm of UCB MSCs in the presence of MSCBM. However, both proteins localized to cell borders when UCB MSCs were grown in either LECBM or LECCM. This localization was lost when extracellular calcium levels were reduced by treatment with EGTA. A second microarray analysis showed that, when UCB MSCs were grown in LECCM instead of LECBM, the relative expression of a subset of genes markedly differed, suggestive of a more HCEC-like phenotype.

**Conclusions:**

Results indicate that UCB MSCs are able to “home” to areas of injured corneal endothelium and that the phenotype of UCB MSCs can be altered toward that of HCEC-like cells. Further study is needed to identify the specific microenvironmental conditions that would permit tissue engineering of UCB MSCs to replace damaged or diseased corneal endothelium.

## Introduction

Restoration of clear vision that was lost due to injury or disease of the corneal endothelium requires either full-thickness corneal transplantation or endothelial keratoplasty. Researchers are currently seeking alternative methods to restore healthy corneal endothelium, since corneas that are considered to be acceptable for transplantation are becoming less available worldwide [1-3]. Tissue bioengineering is an exciting new approach to develop treatments for patients who have lost visual acuity due to corneal endothelial cell injury or disease. One method being investigated is to use cultured donor human corneal endothelial cells (HCEC) to develop bioengineered constructs. HCEC have a finite, donor age-dependent ability to divide [4,5] and the number of times HCECs can be passaged in culture limits the available number of healthy cells for use in these constructs. Researchers are also developing methods to selectively isolate HCEC with characteristics of “young” cells for use in bioengineering [6], while others are testing the use of immortalized HCEC for longer-term cultivation [7], although use of immortalized HCEC for human transplant is problematic. Another possibility is to identify, isolate and culture corneal endothelial stem cells; however, only preliminary evidence currently exists to suggest that there is a population of adult stem cells that gives rise to corneal endothelium [8,9].

The current studies explore the feasibility of altering the phenotype of non-hematopoietic umbilical cord blood mesenchymal stem/stromal cells (UCB MSCs) toward that of HCEC-like cells. This idea is based on the fact that, during eye development in many species, including humans, corneal endothelial cells differentiate from neural crest-derived periocular mesenchymal cells that migrate between the surface epithelium and lens placode [10-15]. Those mesenchymal cells closest to the anterior surface of the developing lens become flattened and establish cell-cell contacts, forming the corneal endothelium. The origin of human corneal endothelium from neural crest-derived mesenchymal cells is supported not only by morphologic studies, but also by immunohistochemical evidence indicating that both cell types express several neural crest proteins, including neuron-specific enolase [16-18], S-100 [19], neural cell adhesion molecule (N-CAM) [16], N-cadherin [20], and vimentin [17,21,22]. Interestingly, during early stages of eye development, differentiation of neural crest-derived mesenchymal cells to form corneal endothelium is strongly influenced by inductive signals produced by lens epithelial cells [20-25].

MSCs offer great promise for use in cell-based therapeutic strategies, primarily because of their intrinsic ability to self-renew and their potential to differentiate into several different cell types [26,27]. MSCs have been isolated from several tissues, including bone marrow [28], adipose tissue [29], synovium [30], skeletal muscle [31], deciduous dental pulp [32], Wharton’s jelly [33], umbilical cord [34], and umbilical cord blood [35]. MSCs isolated from bone marrow and umbilical cord blood have been the most widely studied. UCB MSCs, isolated following full-term deliveries, exhibit stem cell-like plasticity and tend to “home” to and attach to areas of injury where they can differentiate into different cell types depending on the specific microenvironment [27]. The current studies also explore the ability of UCB MSCs to “home” to areas of endothelial cell injury using an ex vivo corneal endothelial wound model, as well as test the ability of three different culture media to alter the UCB MSC phenotype toward that of HCEC-like cells.

UCB MSCs have several advantages over other stem cell sources for the following reasons: 1) Their relative plasticity overcomes the moral and ethical issues of using embryonic stem cells; 2) MSCs from umbilical cord blood are a “younger” type of stem cell than other sources, such as bone marrow, which can exhibit a decrease in both proliferative and differentiation capacity with donor age; and 3) UCB appears to contain populations of MSCs with broader differentiation potential [36] compared to adult mesenchymal stem cells, such as cells isolated from bone marrow. Prior studies by members of our group [37] demonstrated phenotypic heterogeneity in MSCs isolated from umbilical cord blood. Based on this work, two UCB MSC clones were chosen for the current study and designated UCB1 and UCB4 MSCs. UCB1 cells are from the same clone as described previously [37]. These cells are small, elongated, and exhibit relatively fast population doubling times. The characteristics of UCB4 MSCs were not described previously; however, the phenotype of these cells is very similar to that of UCB2 cells, which have been described [37]. These cells are larger and more flattened than UCB1 cells. They grow in focal patches and exhibit slower population doubling times than UCB1 MSCs. Both populations of cells express the following MSC surface markers: CD29 (integrin, beta-1), CD44 (CD44 molecule, Indian blood group), CD73 (5’-nucleotidase, ecto), CD90 (THY1 cell surface antigen), and CD105 (endoglin), as well as COL1A1 (collagen type 1, alpha 1) and FN1 (fibronectin). Neither population exhibits the hematopoietic markers: CD11b (integrin, alpha M), CD34 (CD34 molecule), CD35 (CR1 complement component (3b/4b) receptor-1), or the endothelial cell marker, CD31 (platelet/endothelial cell adhesion molecule-1). Both populations are capable of adipogenic, osteogenic, and chondrogenic differentiation, although they appear to exhibit quantitative differences in differentiation potential [37].

The current studies used microarray analysis to obtain baseline information regarding the relative gene expression of UCB MSCs compared with HCEC. A tissue culture model and an ex vivo corneal endothelial wound model were used to compare the ability of three culture media to alter the phenotype of UCB MSCs toward more HCEC-like cells. The ex vivo wound model was also used to test the ability of UCB MSCs to ‘home” to damaged endothelium. A second microarray analysis identified changes in the expression of UCB MSC genes toward that of HCEC-like cells following differential medium incubation.

## Methods

### Umbilical cord blood samples

De-identified umbilical cord blood (UCB) samples were harvested at birth from full-term deliveries, after informed parental consent, under an Institutional Review Board-approved protocol to the New Jersey Cord Blood Bank located within Coriell Institute for Medical Research, Camden, NJ. After removal of the placenta, blood was collected within the first 10 min of delivery and drained from the distal end of the umbilical vein by gravity into a plastic bag containing 25 ml of citrate phosphate dextrose anticoagulant solution (Medsep Corporation, Covina, CA). These anonymous UCB samples were stored at room temperature and units considered non-bankable due to low volume were processed within 24 h of delivery.

### Umbilical cord blood mesenchymal stem cells (UCB MSCs)

MSCs were isolated from umbilical cord blood and individual clones were cultured and characterized as described previously [37]. Some UCB1 MSCs were labeled by transduction with a self-inactivating (SIN) lentivirus vector expressing enhanced Green Fluorescent Protein (E-GFP) [38] and FACS-sorted to enrich for the GFP-labeled population. Under an appropriate Materials Transfer Agreement and written approval from the Schepens Institutional Review Board, cells were used for further studies. Clones designated as UCB1 and UCB4 were used for the current studies and were carefully thawed and then cultured according to published protocols [37]. Briefly, cells were grown at 37 °C in a humidified atmosphere containing 5% CO_2_ in culture medium, designated for these studies as MSC basal medium (MSCBM). This medium consisted of Dulbecco’s modified Eagle’s Medium with low glucose (Invitrogen Life Technologies, Carlsbad, CA), 10% fetal bovine serum (FBS; Sigma-Aldrich, St. Louis, MO), and 1% penicillin-streptomycin (Invitrogen). Cells were cultured for 3 weeks to obtain sufficient cells for experimental use, while preventing senescence due to multiple passages.

In some experiments, UCB MSCs were seeded onto Permanox tissue culture slides (Sigma-Aldrich) and grown in culture medium until cells filled the slide area. Cells were either directly visualized by phase-contrast microscopy or fixed for 10 min in ice-cold 100% methanol, stained for 10 min with 0.5% crystal violet in methanol at room temperature, and washed with water before visualization with a Nikon Eclipse Inverted Microscope TS100 (Nikon Instruments Inc., Melville, NY). In other experiments, UCB MSCs were grown in various culture media as described and then directly processed for immunocytochemical localization studies or were removed from the culture plate and either processed for microarray analysis or used in ex vivo corneal endothelial wound studies (see below).

### Human corneas

Human corneas were obtained through the National Disease Research Interchange (NDRI, Philadelphia, PA). Handling of donor information by the source eye bank, NDRI, and this laboratory adhered to the tenets of the Declaration of Helsinki in protecting donor confidentiality. All corneas were preserved in Optisol-GS (Bausch & Lomb, Rochester, NY) at 4 °C. Exclusion criteria have been previously published [5]. Briefly, all corneas used in these studies were considered unsuitable for transplant, but retained endothelium with a density of at least 2,000 cells/mm^2^. Corneas were not accepted for study if donor blood samples were not available for serology testing, if detects were microscopically visible in any layer of the cornea, or if gutatta were visible. Corneas were rejected if the time between death and preservation was greater than 24 h, if the donor had diabetes, glaucoma, sepsis, or ocular infection, or had been on large doses of chemotherapeutic agents. Some corneas were used for ex vivo corneal endothelial wound studies (see below). For other studies, the endothelium and Descemet’s membrane were carefully dissected from the corneas and HCEC were cultured as previously described [5,39]. Briefly, under a dissecting microscope, the endothelium with Descemet’s membrane was carefully dissected from the cornea in small strips. Endothelial pieces were incubated overnight in culture medium containing 8% fetal bovine serum to stabilize the cells. The strips were then placed in 0.02% ethylenediaminetetraacetic acid (EDTA) in Hank’s balanced salt solution at pH 7.4 and incubated 1 h at 37 °C to loosen cell-cell junctions. Following incubation, junctions were disrupted by moving the tissue and medium multiple times through the narrow opening of a flame-polished glass pipette. Cells were then collected by centrifugation and cultured at 37 °C in a 5% carbon dioxide, humidified atmosphere.

### RNA isolation

Total RNA was isolated and purified from cultured UCB MSCs and HCECs using an RNAeasy Mini Kit (Qiagen, Valencia, CA). Following quantification of the RNA using a Nanodrop spectrophotometer (Thermo Fisher Scientific, Wilmington, DE), samples were frozen until further analysis.

### Microarray analysis

Microarray experiments were conducted on a GeneChip Human Exon 1.0 ST platform (Affymetrix, Santa Clara, CA). Hybridization and scanning were performed according to manufacturer’s protocols in the Nucleotide and Protein Core Facility at Children’s Hospital of Philadelphia, Philadelphia, PA, similar to our previous studies [37]. Data processing and analysis were performed within the R statistical environment. Affymetrix probes were re-grouped into unique Entrez gene IDs using custom library files downloaded from the BRAINARRAY database. Raw data were normalized and summarized by the RMA (Robust Multichip Averaging) method to generate an N*M matrix, where N is the number of unique Entrez genes and M is the number of samples. The normalized data were log2-transformed for statistical analysis. The hierarchical clustering and heat map plots were generated by the R *hclust* and *heatmap* functions, respectively.

### Quantitative real-time PCR

For quantitative real-time PCR (qPCR) analysis, cDNA was synthesized from total RNA using Super Script III (Invitrogen). Primers specific for *NCAM1*, *TFAP2B* (transcription factor activating enhancer binding protein 2-beta), *CDK15* (cyclin-dependent kinase-15), *MEIS1* (meis homeobox-1), *COL8A1* (collagen type VIII alpha-1), *THY1* (thy1 cell surface antigen), *CDH2* (cadherin-2, type 1 N-cadherin), and *TJP1* (tight junction protein-1 ZO1) were used for analysis and are listed in Table 1. The primers were designed with Primer Express 1.5a software (Invitrogen). A Step One Real Time PCR System (Invitrogen) was used. The PCR reaction was performed in a 25 µl final volume, which contained SYBR Green PCR Master Mix, 6 µM each of human specific primers, and cDNA. Thermal cycling was performed for 40 cycles at 95 °C for 15 s, 60 °C for 1 min and the amount of PCR product was estimated using a relative standard curve quantification method. Melting curve analysis controlled the quality of the PCR products. All samples were analyzed in triplicate and results were normalized to the housekeeping gene, *GAPDH* (glyceraldehyde-3-phosphate dehydrogenase). The data were expressed as mean±SD and shown relative to the highest value (0 to 1). Statistical analysis was performed with an unpaired student *t*-test and differences with a probability (p) value ≤0.05 was considered statistically significant.

**Table 1:** Primers Used for Real Time RT–PCR.

**Target Genes**	**Sequence**	**Product Size (bp)**
*NCAM1*	F: 5′-GACTGTCACTCATTCTCCGATCAG-3′	82
* *	R: 5′-GTATGATTATTTTTGCAGAATTGTTTCC-3′	* *
*TFAP2B*	F: 5′-TTGGGTACATTTGCGAAACG-3′	68
* *	R: 5′-TGTGTGCTGCCGGTTCAA-3′	* *
*CDK15*	F: 5′-CACCCAGCCCAGTTTAGCAA-3′	73
* *	R: 5′-AAATACAGCCCTGGAAGAACCTT-3′	* *
*MEIS1*	F: 5′-GACAACCTCGCCTGTGATTGA-3′	70
* *	R: 5′-CCCCTCAGACCCAACTACCA-3′	* *
*COL8A1*	F: 5′-GGAGATGCCCCACTTGCA-3′	72
* *	R: 5′-GCCGGTTGAATTTCCTTCATAT-3′	* *
*THY-1*	F: 5′-ACCTCTTCCTCTTCCCTGACTTC-3′	78
* *	R: 5′-GCCCAGTGTGCAGTCATTAGC-3′	* *
*CDH2*	F: 5′-GAGCAGTGAGCCTGCAGATTTT-3′	81
* *	R: 5′-TGCTCAGAAGAGAGTGGAAAGCT-3′	* *
*TJP1*	F: 5′-AAAGGAGAGGTGTTCCGTGTTG-3′	98
* *	R: 5′-CGTTCTACCTCCTTATGATTTTTACCA-3	* *
*GAPDH*	F: 5′-GTATCGTGGAAGGACTCATGACCA-3′	126
* *	R: 5′-TAGAGGCAGGGATGATGTTCTGGA-3′	* *

The primers were designed with Primer Express 1.5a software (Applied Biosystems). F: forward primer, R: reverse primer.

### Ex vivo corneal endothelial wound models

Two models using human donor corneas were employed in the current studies. One model consisted of a mechanical scrape wound made in the endothelium in an X-shaped pattern using a capsule polisher (Alcon, Fort Worth, TX). In this model, endothelial cells were removed from the wounded area without damage to the underlying Descemet’s membrane. An endothelial crush wound model was prepared by lightly scraping the endothelial surface with the capsule polisher, leaving the damaged cells on the surface of Descemet’s membrane. Depending on the needs of the experiment, GFP-labeled or unlabeled UCB1 MSCs were grown to confluence in MSCBM and then treated with trypsin-EDTA to remove them from the culture dish. A portion of the cells were incubated with trypan blue and counted using a Cellometer Auto T4 cell counter (Nexcelom Bioscience LLC, Lawrence, MA) to determine the number of viable cells. Wounded corneas were placed endothelial-side up on top of a Costar 12 mm Snapwell insert with a 0.4 μm membrane (Sigma-Aldrich). The base of the corneas was placed in MSCBM. An average of 5×10^5^ viable MSCs per 400 μl volume were then added to the endothelial surface in two applications. First, 200 μl of cells were carefully added to the endothelial side of each cornea and the corneas incubated at 37 °C for 1 h to permit the MSCs to settle onto the wounded endothelial surface. At the end of the incubation period, the medium was gently removed from the corneal cup, the 200 μl containing the remaining cells was added, and the corneas then incubated overnight at 37 °C in a humidified atmosphere in the presence of 5% CO_2_. The following morning, the corneas were removed from the insert and gently placed in conventional tissue culture wells containing the desired culture medium and incubated at 37 °C. Medium was changed 3 times per week and corneas were incubated for 14 days before processing for immunocytochemistry (see below). Cuts were made in the sclera for orientation of the tissue at the end of the experiment.

### Preparation of lens epithelial cell-conditioned medium

SV-40 transformed human lens epithelial cells were obtained from the American Type Culture Collection (ATCC) and cultured in growth medium containing ATCC-formulated Eagle’s Minimal Essential Medium, 20% FBS, and antibiotic/antimycotic solution diluted 1:100. This medium formulation was designated for these studies as lens epithelial cell basal medium (LECBM). Prior to use, a western blot analysis was performed (data not shown) to verify the identification of these cells by testing their ability to synthesize β- and γ-crystallins, as indicated by the ATCC. Cells were grown to confluence in LECBM. Medium was changed twice a week. At each medium change, the spent medium was collected, centrifuged to remove cells and debris, and the supernatant refrigerated until use. This medium was designated as lens epithelial cell-conditioned medium (LECCM). In all experiments, this conditioned medium was diluted 1:1 in LECBM before use.

### Immunocytochemical localization

Immunocytochemical localization studies were conducted according to previously published methods [40]. Briefly, samples were washed in phosphate-buffered saline (PBS), fixed for 10 min in 100% methanol at −20 °C, washed in PBS, and then incubated for 10 min in blocking buffer consisting of 4% BSA diluted in PBS. Samples were then incubated overnight at 4 °C in rabbit polyclonal anti-ZO1 (Invitrogen) diluted 1:150 and/or in mouse monoclonal anti-N-cadherin (BD Transduction Laboratories, San Diego, CA) diluted 1:50. After washing, samples were incubated for 1 h in rhodamine-conjugated donkey anti-rabbit IgG and/or in FITC-conjugated donkey anti-mouse IgG (Jackson ImmunoResearch Laboratories, West Grove, PA) diluted 1:100. All antibodies were diluted in blocking buffer. To visualize all nuclei, samples were incubated for 15 min at room temperature in iodide (TO-PRO3: Invitrogen) diluted 1:1,000 in PBS. Negative controls consisted of samples incubated in secondary antibody only. Following washing, corneas were placed endothelial-side up in tissue mounting medium (Vectashield: Vector Laboratories, Burlingame, CA) and covered with glass coverslips. Cultured UCB MSCs were mounted in the same medium before addition of glass coverslips. Fluorescence staining was visualized using a Leica TSC-SP2 confocal microscope (Bannockburn, IL). A Z-series was captured with a step size of 0.5 μm per image. Z-series images were then collapsed onto a single image plane by projecting the maximal pixel intensity of the images. Each experiment was conducted 2–3 times to test for consistency of the results.

## Results

### Morphological characteristics of two UCB MSC populations

**Figure 1:**
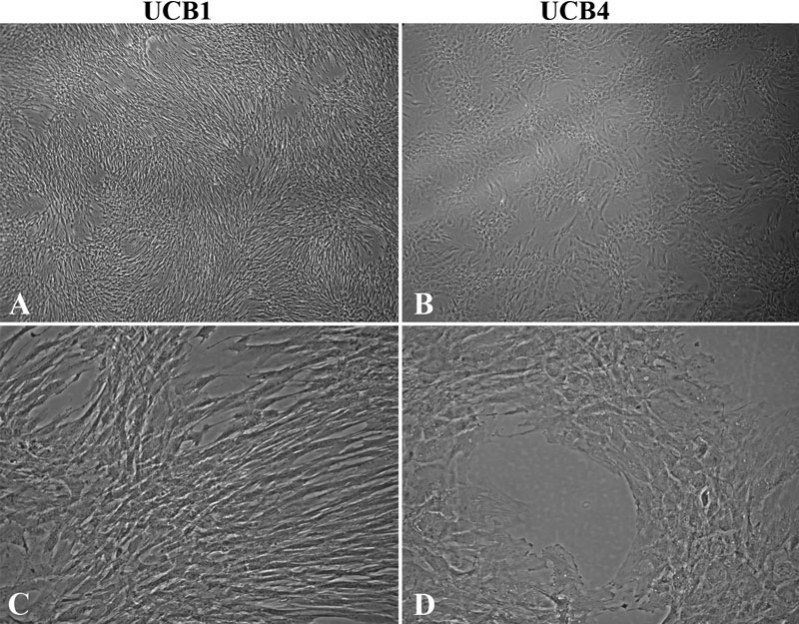
Phase-contrast images of UCB1 and UCB4 MSCs. Note the more elongated shape and swirl pattern formed by UCB1 cells compared with the broader shape of UCB4 cells growing in focal patches. UCB1 MSCs are passage 11. UCB4 MSCs are passage 3. Original magnification of (**A**) and (**B**): 4×. Original magnification of (**C**) and (**D**): 10×.

Two different populations of UCB MSCs, designated UCB1 and UCB4, were used in the initial stages of these studies. Both UCB1 and UCB4 MSCs were grown in MSCBM until cells covered the tissue culture dish. As can be seen by the phase-contrast images in Figure 1, both types of MSCs exhibited a generally spindle-shaped morphology; however, UCB1 cells were more elongated and fibroblast-like, while UCB4 cells were larger and more flattened. At high density, UCB1 cells were relatively closely associated and made a swirl-like pattern of overlapping cells, whereas, UCB4 cells grew in focal patches.

### Comparison of gene expression in UCB MSCs and HCEC

**Figure 2:**
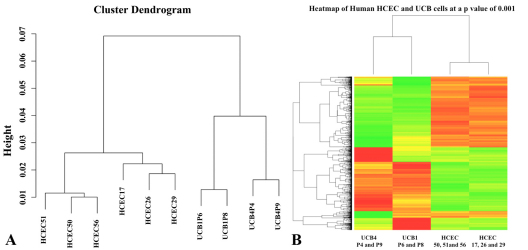
Cluster dendrogram and heat map results. Cluster dendrogram (**A**) shows relationships in gene expression among UCB1, UCB4, young HCEC, and older HCEC samples. The heatmap in (**B**) shows relative levels of gene expression at a p-value of 0.001 between UCB1 and UCB4 MSCs and HCECs from young and older donors. The relative levels of gene expression are depicted using a color scale where red represents the lowest and green represents the highest level of expression.

Microarray analysis was used to compare gene expression between MSCs and HCEC. The cluster dendrogram in Figure 2A indicates relative similarity in gene expression among closely related groups, i.e., among young HCEC, older HCEC, UCB1, and UCB4 samples. As expected, relative gene expression in both sets of HCEC samples exhibited closer similarity to each other than to the two sets of UCB samples. The same is true of the two types of UCB samples versus HCEC. Significance Analysis of Microarrays (SAM) [41] was conducted to compare relative gene expression between UCB1 and UCB4 MSCs, each from two different passages (UCB1P6, UCB1P8, UCB4P4, and UCB4P9) and passage 2 HCECs isolated and cultured from three young (17, 26, and 29 years old) and three older donors (50, 51, and 56 years old). This comparison was made to establish a baseline of relative gene expression for UCB MSCs and HCECs before attempting to differentiate MSCs toward more HCEC-like cells. A list of the top 250 genes in which the mean expression level in UCB MSCs is significantly higher than in HCEC is presented in Appendix 1. Appendix 2 contains a list of the top 250 genes in which the mean expression level in UCB MSCs is significantly lower than in HCEC. Figure 2B presents a heat map comparing the relative expression of individual genes at an ANOVA p-value of 0.001. The heat map indicates significant differences in the relative level of individual gene expression among the UCB1, UCB4, young HCEC, and older HCEC samples. In general, UCB samples exhibited greater similarity in expression to each other than to either group of HCEC and vice versa.

**Figure 3:**
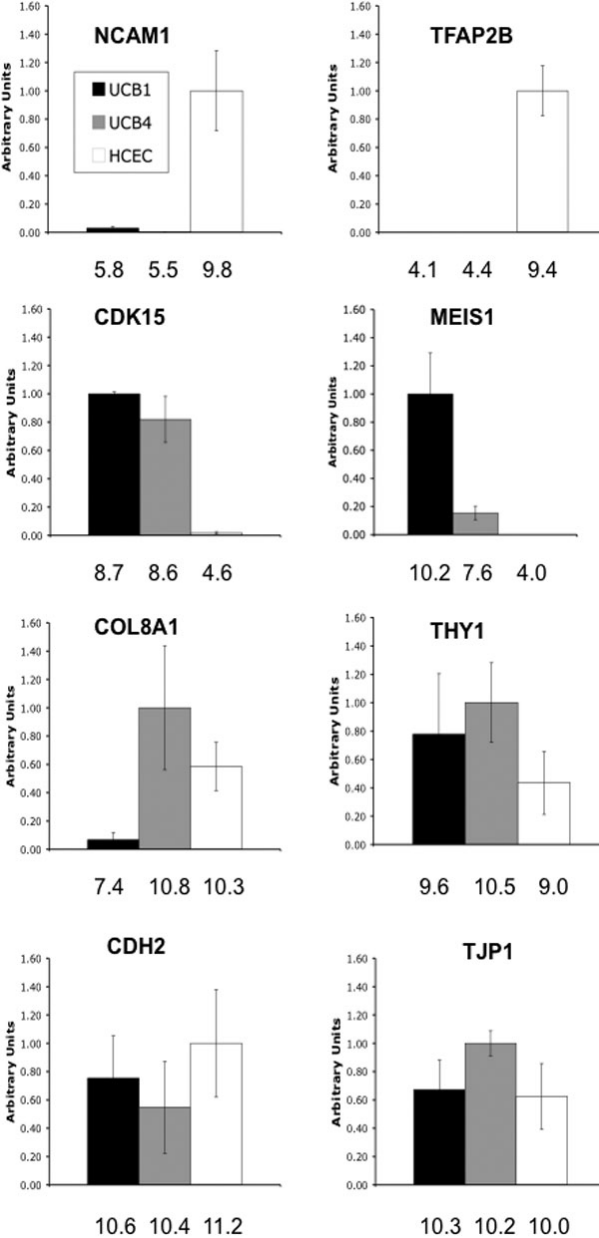
Quantitative real-time PCR confirms differences in gene expression identified by microarray analysis in UCB1, UCB4, and HCEC. Relative expression levels were normalized to the housekeeping gene *GAPDH* and are shown relative to the highest value (0 to 1). Error bars represent one standard deviation for the four UCB (two UCB1 and two UCB4) and six HCEC biologic replicates tested. Robust multi-array average (RMA) estimated expression levels from the Affymetrix array, averaged for the biologic replicates within each cell type, are listed on the x-axis.

Quantitative real time-PCR studies were then conducted using RNA isolated from the same UCB MSC and HCEC samples to verify results of the microarray analysis for a subset of genes. For these studies, data was averaged and comparison was made between UCB1 MSCs, UCB4 MSCs, and HCECs from both young and older donors (Figure 3). As observed in the microarray analysis, HCECs expressed significantly higher mRNA levels of both *NCAM1* (neural cell adhesion molecule-1) and *TFAP2B* (transcription factor AP-2, beta) compared with either type of MSC. Both UCB1 and UCB4 MSCs expressed significantly higher mRNA levels of *CDK15* (cyclin-dependent kinase-15) and *MEIS1* (Meis homeobox1), as indicated by the microarray analysis. The relative expression of four additional mRNAs was tested. *COL8A1* (collagen VIII, alpha-1) is expressed in corneal endothelial cells [42], as well as in UCB1 MSCs [37]. Results from qPCR indicated that, although all samples were positive, UCB1 MSCs expressed relatively low, but detectable, levels of *COL8A1* in comparison with either UCB4 or HCECs. The cell surface antigen, THY1 (CD90), is an important marker of non-hematopoeitic UCB MSCs [37,43]. The qPCR results indicated that *THY1* mRNA is not only expressed in UCB1 and UCB4 MSCs, but also in HCEC, with expression in HCEC being somewhat lower than in the MSCs. CDH2 (cadherin-2 / N-cadherin]) is expressed in corneal endothelial cells [44]. Expression of this cadherin isoform has also been detected in MSCs [45]. Results of the qPCR showed a relatively similar level of N-cadherin mRNA expression in the two UCB MSCs and HCEC samples. TJP1 (tight junction protein-1 / ZO1) is expressed in corneal endothelial cells and forms a discontinuous immunolocalization pattern at cell-cell borders [46,47]. This protein has also been detected in MSCs [45]. Comparisons for *ZO1* mRNA indicated a similar expression in the two types of MSC samples tested, as well as in HCECs.

Following these microarray and q-PCR analyses, it was decided to use only UCB1 MSCs for subsequent studies. This decision was based on the overall gene analysis results, which showed somewhat closer gene expression levels in UCB1 to HCECs compared with UCB4, and on the fact that previous studies [37] suggested that UCB1 MSCs exhibit a greater differentiation potential than UCB4 cells.

### Effect of culture media on the morphology of UCB1 MSCs in tissue culture

**Figure 4:**
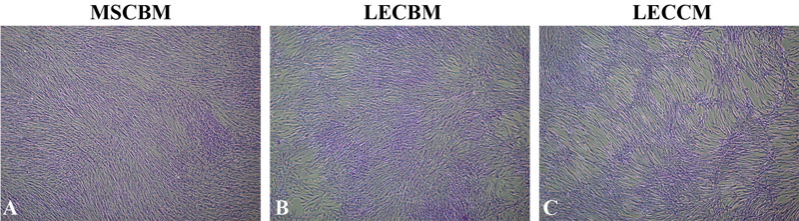
Crystal violet-stained light microscopic images of UCB1 MSCs. Cells grown in MSC basal medium (MSCBM; **A**), lens epithelial cell basal medium (LECBM; **B**), or lens epithelial cell-conditioned medium (LECCM; **C**) show relative differences in cell shape and culture characteristics. Original magnification: 4×.

During corneal development, appropriate differentiation of neural crest mesenchymal cells to form corneal endothelium appears to be dependent on inductive signals from the lens [20-25]. For this reason, we tested whether soluble factors from cultured lens epithelial cells would affect the differentiation of UCB1 MSCs toward more HCEC-like cells using lens epithelial cell-conditioned medium (LECCM) prepared as indicated above. LECCM, the basal medium used for growth of lens epithelial cells (LECBM), and the basal medium used for culture of MSCs (MSCBM) were first compared for their effect on the morphology of UCB1 MSCs in culture. Crystal violet-stained images of UCB1 MSCs grown in the 3 culture media are shown in Figure 4. As described previously [37] and shown in Figure 1A,C, UCB1 MSCs grown in MSCBM (Figure 4A) exhibited elongated, fibroblastic-like shapes and tended to form a swirl pattern with evidence of cellular overlap. MSCs grown in LECBM (Figure 4B) also exhibited elongated, fibroblastic-like shapes, but the cultures tended to have more open areas between cell clusters where cells appeared to be somewhat more broad and flattened. Cells within clusters formed a random criss-cross pattern, indicating multiple cell layers. UCB1 MSCs grown in LECCM (Figure 4C) were consistently more highly elongated than cells grown in the other two media. Cells in the more open areas of the culture tended to form parallel arrays, while cells located within clusters showed evidence of multi-layering.

### Effect of culture media on junction formation in UCB1 MSCs in tissue culture

**Figure 5:**
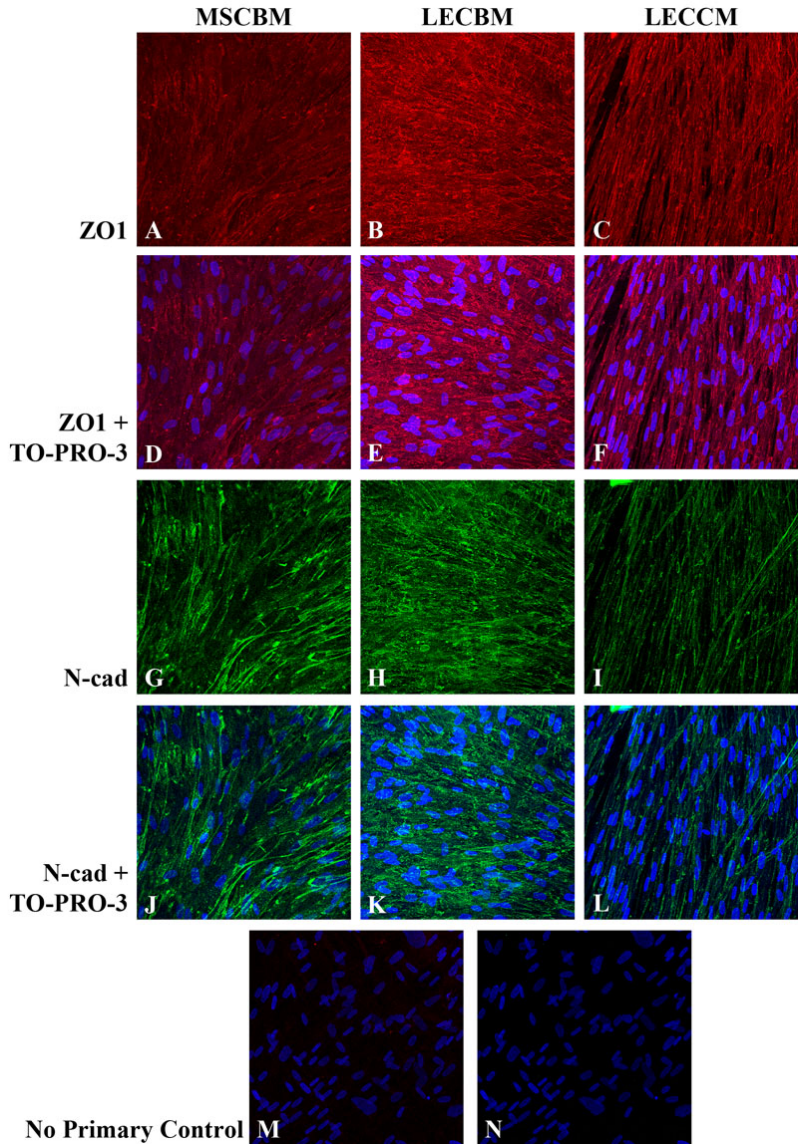
ZO1 (red) and N-cadherin (green) staining patterns in UCB1 MSCs incubated in three different culture media. Images in **A**-**C** show ZO1 staining alone, while images **D**-**F** show an overlay of the ZO1 and TO-PRO-3 (blue) staining, so individual cells can be observed. Images **G**-**I** show N-cadherin staining alone, while images **J**-**L** show an overlay of the N-cadherin and TO-PRO-3 staining. Images **M** and **N** are negative controls showing overlays of the rhodamine and TO-PRO-3 channels (**M**), and FITC and TO-PRO-3 channels (**N**). Original magnification: 40×.

Immunolocalization studies were conducted to determine the effect of the three culture media on the subcellular localization of the tight junction-associated protein, ZO1, and the adherens junction protein, N-cadherin, since both these proteins are localized at cell-cell borders in HCEC and help support the barrier function of these cells [44,46]. For these studies, UCB1 MSCs were grown in each of the three types of media until cells filled the culture dish and then immunostained for both ZO1 and N-cadherin. Figure 5 presents images of the cells stained for either ZO1 or N-cadherin alone to clearly show the staining pattern, as well as the same images showing TO-PRO-3 staining to indicate the presence of nuclei. Growth in MSCBM resulted in a generally diffuse cytoplasmic localization of ZO1 (Figure 5A,D), although a few intensely stained short, linear plaques could be seen within the cytoplasm and, in some cells, these plaques appeared to be localized to the lateral plasma membrane. MSCs grown in LECBM (Figure 5B,E) exhibited a light, diffuse cytoplasmic stain for ZO1, but under this medium condition, the relative number and intensity of the short, linear plaques were increased at cell borders. Growth of UCB1 MSCs in LECCM (Figure 5C,F) showed a similar increase of the size and relative number of positive ZO1 plaques at cell borders. The LECCM-induced cellular elongation and parallel arrangement were easily visualized by the ZO1 immunostaining. As with ZO1, the staining pattern for N-cadherin differed based on the growth medium. In UCB1 MSCs grown in MSCBM (Figure 5G,J), N-cadherin showed mainly a diffuse cytoplasmic localization with occasional linear staining at cell borders. Cells grown in LECBM (Figure 5H,K) showed light, diffuse cytoplasmic staining, but there was also more intense staining in linear plaques at cell borders. MSC grown in LECCM (Figure 5I,L) showed positive staining for N-cadherin mainly along cell borders. Images in Figure 5M,N show the negative controls for ZO1 and N-cadherin, respectively, in which cells were only incubated in the presence of secondary antibody. The lack of rhodamine or FITC staining in these images indicates the overall specificity of the staining results using primary antibody.

**Figure 6:**
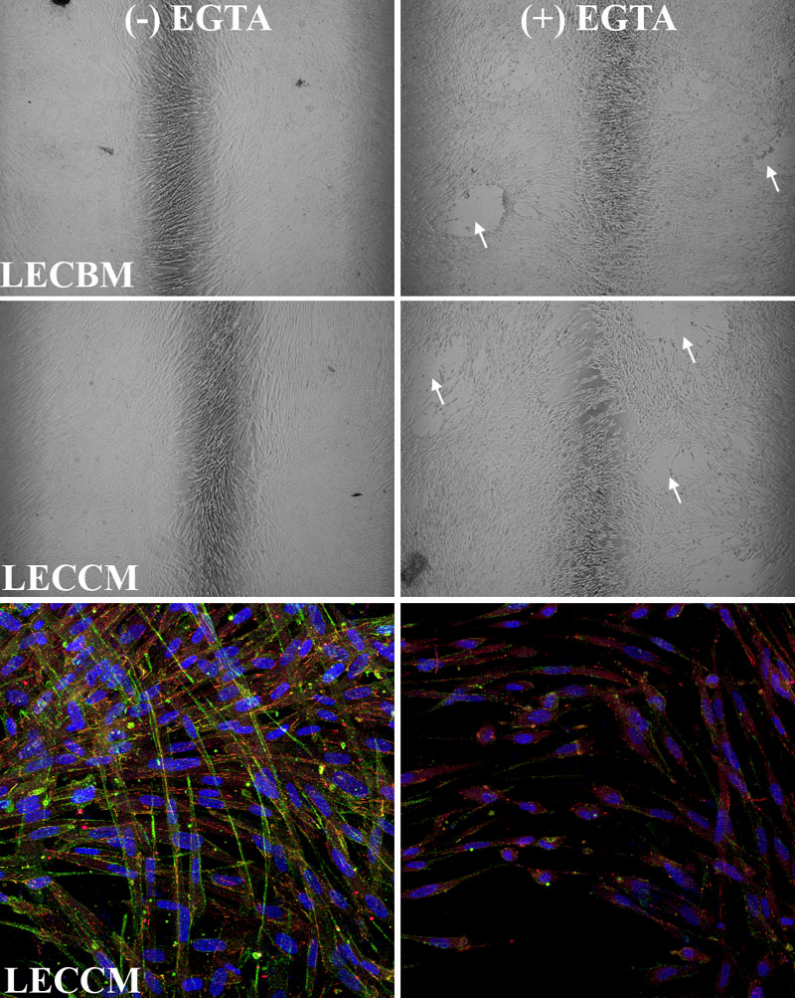
Effect of EGTA treatment on UCB1 MSC morphology and junction-associated protein localization. Top four phase-contrast images demonstrate that EGTA treatment induces separation of UCB1 MSCs in cultures grown in either lens epithelial cell basal medium (LECBM) or lens epithelial cell-conditioned medium (LECCM). Arrows in the (+) EGTA images indicate large spaces between cells. Confocal fluorescence images at the bottom demonstrate changes in the relative localization of N-cadherin (FITC) and ZO1 (rhodamine) in UCB1 MSCs grown in LECCM. Both bottom images are overlays with TO-PRO-3 (blue) to visualize nuclei. Phase contrast original magnification: 4×. Confocal original magnification: 40×.

Overall, results indicated that both ZO1 and N-cadherin proteins are expressed by UCB1 MSCs under each of the culture medium conditions tested; however, the subcellular localization of these proteins differed with the specific medium. Cells grown in the presence of LECBM and, particularly in the presence of LECCM, showed increased localization of both proteins at cell-cell borders, suggesting the formation of tight and adherens junctions between cells. Since the integrity of both tight [48] and adherens junctions [49] is dependent on the presence of extracellular calcium, we tested the effect of removal of extracellular calcium on the morphology of UCB1 MSCs and the localization of ZO1 and N-cadherin. For these studies, UCB1 MSC, grown in LECBM or LECCM, were incubated in the presence and absence of a 5 mM concentration of the calcium chelator, ethylene glycol tetraacetic acid (EGTA). The relative integrity of the resulting cultures was examined by phase-contrast microscopy. As shown in Figure 6, UCB1 MSCs grown in either LECBM or LECCM and treated with EGTA showed large breaks within the culture and areas where individual cells were located some distance from each other. This pattern strongly suggested that treatment with EGTA had caused the separation and loss of cells within the culture. The effect of EGTA treatment on ZO1 and N-cadherin localization in cells grown in LECBM and LECCM was determined using fluorescence confocal microscopy. Similar results were obtained with cells grown in both media. Images in Figure 6 show that EGTA-treated UCB1 MSCs grown in LECCM lost their highly elongated shape and became separated from each other within the cell sheet. The relative intensity of both ZO1 and N-cadherin in the EGTA-treated cultures was greatly reduced compared with untreated controls and the staining patterns changed from the proteins being mainly localized to cell borders in non-EGTA-treated cultures to being diffusely distributed within the cytoplasm in cultures treated with EGTA. This change in subcellular localization is consistent with the loss of cell-cell junctions.

### UCB MSCs attach to wounded endothelium in ex vivo cornea models

**Figure 7:**
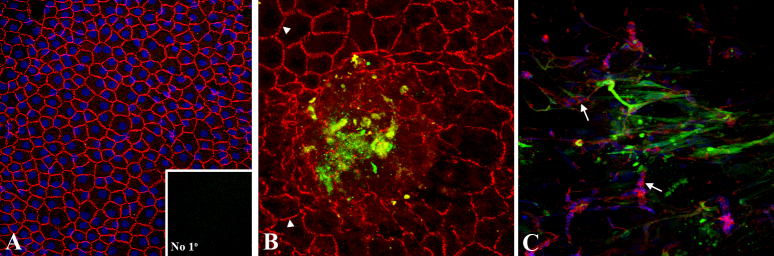
Attachment of GFP-labeled UCB1 MSCs to damaged endothelium. The image in (**A**) shows lack of attachment of UCB1 MSCs to unwounded endothelium. The inset shows results of the no-primary negative control for ZO1 staining. GFP-labeled UCB1 MSCs in (**B**) attached to damaged endothelium in the crush wound model. Arrowheads indicate the ZO1 pattern of unwounded HCEC. The image in (**C**) shows attachment of GFP-labeled UCB1 MSCs to remnants of damaged endothelium in the scrape wound model. Arrows indicate areas of damaged HCEC. Red: ZO1. Blue: TO-PRO-3-stained nuclei. Blue: TO-PRO-3-stained nuclei. Original magnification: 40×.

The feasibility of using UCB1 MSCs to replace HCEC that have been lost due to mechanical trauma was determined by testing the ability of these cells to “home” to wounded corneal endothelium in donor human corneas. Two types of ex vivo corneal endothelial wound models were tested. These included a crush wound in which endothelial cells were damaged with a capsule polisher and a scrape wound in which endothelial cells were scraped off Descemet’s membrane, but leaving Descemet’s membrane intact. After wounding of the endothelium, green fluorescent protein (GFP)-labeled UCB1 MSCs were seeded onto the endothelial surface and then maintained in MSCBM. Ex vivo corneas containing intact endothelium were used as controls. After 2 weeks in culture, the relative attachment of GFP-labeled UCB1 MSCs was evaluated by fluorescence confocal microscopy. ZO1 immunostaining was used to identify undamaged HCEC, because of its distinctive staining pattern in corneal endothelium [46,47]. Damaged HCEC were identified by the presence of ZO1 staining, but loss of this distinctive pattern. Nuclei were detected by TO-PRO-3 staining. UCB1 MSCs did not attach to undamaged endothelium (Figure 7A), but consistently attached to damaged endothelium in both the crush wound (Figure 7B) and scrape wound models (Figure 7C). Relatively little attachment of UCB1 MSCs to denuded Descemet’s membrane was observed (Figure 7C). Overall, these results indicate that UCB1 MSCs can participate in the healing of corneal endothelial wounds by attaching to damaged HCEC in ex vivo corneas. The crush wound model was chosen for all subsequent ex vivo studies, because it best parallels conditions expected for the healing of corneal wounds resulting from mechanical trauma.

### Effect of culture media on UCB1 MSCs in the ex vivo crush wound model

**Figure 8:**
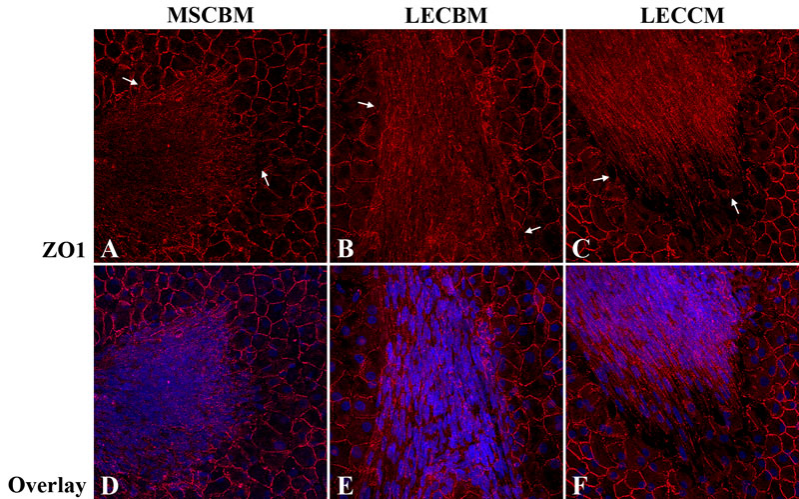
Effect of 3 culture media on UCB1 MSC association with damaged endothelium. Fluorescence confocal microscopic images show the formation of MSC cell sheets in areas of damaged HCEC (arrows in **A**-**C**). Note that, in wounded corneas incubated in MSCBM, ZO1 is localized diffusely within the cytoplasm of the MSCs. In wounded corneas incubated in LECBM or LECCM, ZO1 tended to be localized at the lateral borders of MSCs. Red: ZO1. Blue: TO-PRO-3-stained nuclei. Original magnification: 40×.

Studies were conducted to determine the effect of the three culture media on association of non-GFP-labeled UCB1 MSCs with damaged endothelium using the ex vivo crush wound model. For these studies, crush wounds were made in the endothelium of donor corneas. Corneas were incubated for 48 h in MSCBM plus 30 µg/ml 5-fluorouracil (5-FU) to inhibit proliferation of the HCEC and prevent premature closure of the wounds. After washing to remove the 5-FU, UCB1 MSCs were placed on the endothelial side of the corneas and incubated for 2 weeks in the presence of MSCBM, LECBM, or LECCM. Corneas were then processed for ZO1 staining and nuclear labeling with TO-PRO-3. As can be seen in Figure 8, UCB1 MSCs associated with damaged HCEC regardless of the culture medium used. However, there was a consistent difference noted in the relative arrangement of the cells based on the culture medium. In the presence of MSCBM (Figure 8A,D), MSCs showed a relatively random orientation within the damaged areas, whereas, when MSCs were incubated in LECBM (Figure 8B,E) or LECCM (Figure 8C,F), the MSCs tended to form a more oriented and more tightly associated cell sheet in the wound area.

**Figure 9:**
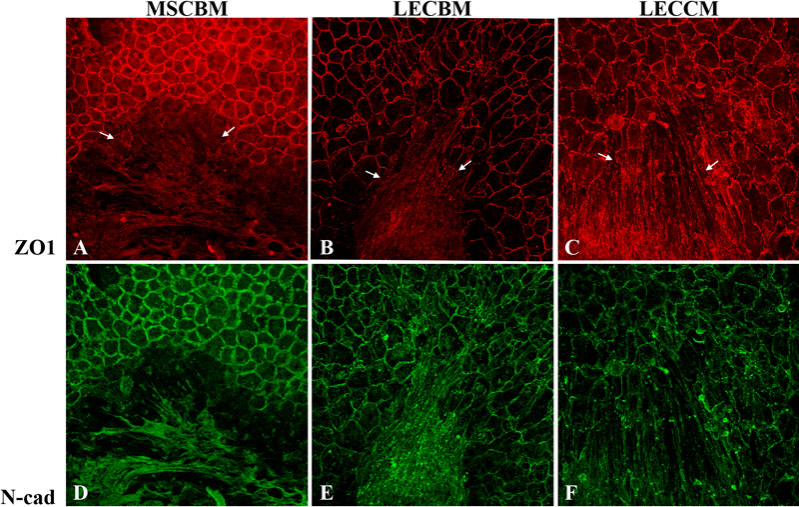
Effect of 3 culture media on ZO1 and N-cadherin localization in MSCs associated with damaged HCEC. Fluorescence confocal microscopic images show that, in wounded corneas incubated in MSCBM, ZO1 and N-cadherin were localized diffusely within the cytoplasm of the MSCs. In wounded corneas incubated in LECBM or LECCM, ZO1 and N-cadherin tended to localize at the lateral borders of MSCs. Arrows in **A**-**C** show edges of the damaged endothelium. Red: ZO1. Green: N-cadherin. Original magnification: 40×.

Experiments were then conducted to determine the effect of the three culture media on the localization of ZO1 and N-cadherin when UCB1 MSCs were added to ex vivo corneal endothelial crush wounds. A similar protocol was used for these studies as for the ex vivo studies discussed above. The only difference was that the tissue was processed for both ZO1 and N-cadherin immunostaining. Images in Figure 9 show that both ZO1 and N-cadherin were expressed under all 3 medium conditions; however, as observed in the tissue culture studies, the relative localization of both proteins differed depending on the culture medium. In UCB1 MSCs incubated in MSCBM (Figure 9A,D), ZO1 and N-cadherin were both diffusely distributed within the cytoplasm. Both proteins showed a greater association with cell borders when MSCs were incubated in LECBM (Figure 9B,E), but the overall arrangement of the cells was relatively random. MSCs incubated in LECCM (Figure 9C,F) showed the greatest association of both ZO1 and N-cadherin at cell borders. Cells also formed a relatively tight sheet of parallel cells within the wound area.

### Effect of culture media on relative gene expression of UCB1 MSCs

Of the several culture media tested using either the tissue culture or ex vivo crush wound models, it was clear that LECCM had the greatest influence on the phenotype of UCB1 MSCs. Because of these changes, it was decided to compare the effect of LECBM and LECCM on relative gene expression in UCB1 MSCs. For these studies, UCB1 MSCs were cultured in the presence of LECBM or LECCM until the cells filled the culture dish. Cells were then washed and RNA was extracted and purified as previously. A second round of microarray analyses was conducted similar to those described above. Careful analysis indicated several differences in relative gene expression. Of the 250 genes that were expressed at higher levels when UCB1 MSCs were grown in LECCM compared with LECBM (Appendix 3), 18 were also expressed at higher levels in HCEC compared with either UCB1 or UCB4 MSCs (compare with Appendix 2). These genes included C3 (complement component 3), IL8 (interleukin 8), PTGS2 (prostaglandin-endoperoxide synthase 2, also called prostaglandin G/H synthase and cyclooxygenase), RAB27B (RAB27B, member RAS oncogene family), CXCL1 (chemokine (C-X-C motif) ligand 1 (melanoma growth stimulating activity, alpha), IL13RA2 (interleukin 13 receptor, alpha 2), TFPI2 (tissue factor pathway inhibitor 2), PLA2G4A (phospholipase A2, group IVA (cytosolic, calcium-dependent), STC1 (stanniocalcin 1), DPP4 (dipeptidyl-peptidase 4), GDF15 (growth differentiation factor 15), AKR1C1 (aldo-keto reductase family 1, member C1 (dihydrodiol dehydrogenase 1; 20-alpha (3-alpha)-hydroxysteroid dehydrogenase), DNER (delta/notch-like EGF repeat containing), SAT1 (spermidine/spermine N1-acetyltransferase 1), DUSP6 (dual specificity phosphatase 6), MTHFD2L (methylenetetrahydrofolate dehydrogenase (NADP+ dependent) 2-like), MANSC1 (MANSC domain containing 1), and RNY5 (RNA, Ro-associated Y5). Together, this data indicates a change in the relative expression of a subset of genes based on incubation in LECCM and differentiation of UCB1 MSCs toward a more HCEC-like phenotype.

## Discussion

Several studies have reported the usefulness of mesenchymal stem cells for the treatment of corneal diseases. For example, UCB MSCs have been transplanted into the corneas of lumican null mice [50]. This treatment significantly improved corneal transparency and increased stromal thickness. There is also a report of the successful use of autologous bone marrow MSCs as a means of replacing corneal endothelium in rabbits in vivo [51].

Although UCB MSCs and HCECs are clearly different cell types, they share several important mesenchymal cell characteristics. The basic hypothesis driving the current studies was that it should be possible to modify the phenotype of UCB MSCs so that they could function as HCECs. As a first step in testing this hypothesis, the current in vitro studies tested the feasibility of altering the phenotype of UCB MSCs toward that of HCEC-like cells and investigated whether UCB MSCs can “home” to sites of corneal endothelial cell injury.

In these studies, two different clones of UCB MSCs were initially examined. The first series of microarray data established a baseline of relative gene expression between MSCs and HCEC that could be used for subsequent comparison following attempts to differentiate UCB MSCs toward HCEC-like cells. As expected, the results showed that UCB1 and UCB4 MSCs exhibit close, but not identical gene expression patterns. Both MSC clones showed a closer gene expression profile to each other than to HCEC. Similarly, the profiles of HCECs from young and older donors were closer to each other than to either of the MSC clones. The analysis also identified certain genes that may act as markers to distinguish between these two cell types and provide evidence of differentiation of MSCs toward HCEC-like cells. Although expression of only a small subset of genes was verified by q-PCR, the results paralleled the findings of the microarray analysis. As indicated above, the choice of UCB1 MSCs for further study was based on information indicating that this clone displayed greater overall differentiation potential [37]. In fact, our previous studies confirm that these cells behave as precursors, recapitulating basic developmental pathways when assayed at the molecular level and used as an in vitro model for somitogenesis, a basic process in musculoskeletal development [52].

In vivo, cell shape and the ability of cells to maintain corneal transparency are major criteria for identification of healthy corneal endothelium. Because authentic phenotypic markers for HCEC have not been identified, in vitro identification of these cells can be problematic. For this reason, a combination of criteria is generally used for cell identification. These include cell shape, the expression and appropriate localization of proteins known to support the barrier and pump functions of these cells, as well as the expression of proteins that help distinguish HCEC from corneal keratocytes and epithelial cells. In the current studies, several approaches were used to initially compare HCEC and UCB MSCs and to follow phenotypic changes in UCB MSCs that would suggest their alteration to more HCEC-like cells.

Differentiation of MSCs along specific lineages is dependent, at least in part, on microenvironmental conditions [45,53]. In these studies, lens epithelial cell-conditioned medium (LECCM) was used as a means of mimicking the effect of the lens during corneal endothelial differentiation from neural crest mesenchymal cells. A major effect of this medium was the consistent elongation of the UCB1 MSCs, both in tissue culture and in the ex vivo crush wound model. This change in cell shape did not occur as consistently or to the same extent when cells were exposed to the basal medium used to culture the lens epithelial cells (LECBM). Overall, these results suggest that the shape of UCB1 MSCs can be altered based on culture medium composition and that medium conditioned by lens epithelial cells contains one or more factors that enhance the elongation of UCB1 MSCs. The specific factor(s) in the medium that induce this change were not identified in these studies. Of interest is the fact that the shape of UCB MSCs, when incubated in LECCM, was similar to that of lens fiber cells. The original microarray analysis conducted in this study indicates a very similar expression of both αA- and αB-crystalline genes in UCB1 MSCs and in HCECs (data not shown), and there was no significant change in the level of either of these genes when MSCs were incubated in LECCM. As such, at least at this level of investigation, there is no strong evidence that MSCs were altered to lens fiber-like cells upon LECCM incubation; however, it would be important to thoroughly test for this possibility in future studies.

It is unclear from the current studies whether the shape of UCB1 MSCs could be modified to more closely resemble that of HCEC. Of interest is the fact that several investigators have observed that HCEC can change from the characteristic flattened, hexagonal shape to that of elongated, more fibroblast-looking cells [54-59]. These alterations in cell shape appear to be due to a form of endothelial-mesenchymal transition mediated by environmental factors such as extracellular matrix composition and specific growth factors. Since endothelial cells themselves can change to an elongated, fibroblastic shape, the use of shape for the identification of HCEC-like cells in these studies was problematic. The original elongated shape of UCB MSCs in their basal medium (MSCBM) was enhanced by incubation in LECBM, and particularly in LECCM. The specific intracellular signals leading to this morphologic change are not known and the scope of the current study did not permit investigation of whether the shape of UCB MSCs could be altered to more closely resemble that of HCEC. Further investigation of the molecular basis of the epithelial-to-mesenchymal transition in HCEC may provide information important to stimulate a mesenchymal-to-epithelial transition of UCB MSCs.

Studies of human bone marrow-derived MSCs [45] have demonstrated the formation of calcium-dependent junctional complexes containing several adhesion-associated proteins, including N-cadherin and ZO1. Since the barrier function of HCEC is dependent, at least in part, on the formation of N-cadherin-containing adhesion junctions [44], as well as on ZO1-associated tight junctions [46], it was important to test their relative expression and subcellular localization in UCB MSCs incubated under different conditions. Microarray and q-PCR analyses indicated that both HCEC and UCB MSCs express N-cadherin and ZO1 at relatively similar levels. Importantly, the immunolocalization studies provided evidence of medium-dependent changes in the localization of these proteins in UCB1 MSCs in both the tissue culture and ex vivo corneal endothelial crush wound models. For these studies, the lack of staining in the negative controls indicated the overall specificity of the results. The primary antibodies used did not specifically distinguish between the cytoplasmic (newly synthesized) and plasma membrane (functional) forms of these proteins, therefore, the localization of both proteins to the cytoplasm, particularly in cells incubated in MSCBM, suggests that the proteins were being synthesized under these conditions, but not transported to the plasma membrane. Incubation in LECBM, and particularly LECCM, resulted in greater localization of both proteins to the plasma membrane. The fact that EGTA treatment increased spaces between the cells and caused a change in localization of both ZO1 and N-cadherin from cell borders to a diffuse distribution within the cytoplasm strongly suggests that true, calcium-dependent junctions were formed when cells were grown in LECBM and LECCM. The consistent movement of both these proteins to the plasma membrane, particularly in UCB1 MSCs grown in LECCM, indicates the induction of an important change toward HCEC-like cells. The molecular basis for this change is not known and should be investigated in future studies. One of the major differences between MSCBM and LECBM is an increase in FBS concentration from 10% to 20%. Although the effect of these media on cell proliferation was not directly determined in this study, the increase in FBS concentration may be responsible for an overall increase in cell density, thereby promoting formation of both tight and adherens junctions between the more closely-packed neighboring cells.

Of significance was the consistent observation that UCB1 MSCs attached with great efficiency to wounded HCEC, but did not adhere strongly to either unwounded endothelium or to Descemets’ membrane that had been scraped to remove HCEC. This tendency of MSC to “home” to wound areas has been observed previously [60-62] and would be important in the treatment of corneal endothelial injuries. Of the three culture media tested, LECCM appeared to promote the closest cell-cell association and, in the ex vivo crush wound model, yielded an apparent monolayer of closely-packed, elongated cells. Incubation in LECCM also appeared to alter the expression of several genes in UCB MSCs to more closely resemble that of HCEC. Although LECCM appeared to have the greatest effect on the UCB MSC phenotype, it did not produce optimal results, indicating the need for additional study to identify the specific microenvironmental factors needed to consistently alter the phenotype of UCB MSCs to that of HCEC-like cells.

Overall, results of these studies strongly indicate that UCB MSCs can “home” to areas of corneal endothelial cell injury, as well as demonstrate the feasibility of altering the phenotype of UCB MSCs toward that of HCEC-like cells. Additional studies, including in vivo testing, are now needed to identify the specific conditions that would best support the ability of UCB MSCs to replace corneal endothelial cells lost due to damage or disease as a means of restoring corneal transparency.

## 

## Appendix 1

Appendix 1. Four microarray/ human gene expression studies: housekeeping and non-housekeeping genes expressed. To access the data, click or select the words “Appendix 1.” This will initiate the download of a compressed (pdf) archive that contains the file.

## Appendix 2

Appendix 2. Non-HK TM genes with altered expression due to glaucoma relevant exprimental manipulations. To access the data, click or select the words “Appendix 2.” This will initiate the download of a compressed (pdf) archive that contains the file.

## Appendix 3

Appendix 3. Non-HK human TM expressed genes grouped based on function. To access the data, click or select the words “Appendix 3.” This will initiate the download of a compressed (pdf) archive that contains the file.
